# Flexural behavior of 3D *para*-aramid/phenolic/nano (MWCNT) composites

**DOI:** 10.1039/c7ra13437a

**Published:** 2018-02-14

**Authors:** K. Bilisik, G. Erdogan, E. Sapancı

**Affiliations:** Department of Textile Engineering, Faculty of Engineering, Erciyes University 38039 Kayseri Turkey kadirbilisik@gmail.com +90 352 437 5784 +90 352 437 4937; ROKETSAN Industries 06780 Elmadag-Ankara Turkey

## Abstract

In this work, the flexure properties of nanostitched and nanoprepreg three dimensional (3D) *para*-aramid/phenolic composites were studied. Four types of composite were developed. They were called stitched/nano, stitched, base/nano and base. The flexure strength and modulus of the stitched/nano composites were slightly improved compared to those of the base composites due to the addition of the stitching yarn and multiwall carbon nanotubes (MWCNTs). The flexure failure of the base and base/nano structures was matrix peeling and large delaminated areas, whereas the stitched and stitched/nano composites had warp deformation and no visible matrix/fiber damage. In addition, the delaminated areas were severely restricted. The results showed that introducing the stitching fiber and multiwall carbon nanotubes in the base structure improved its out-of-plane failure properties as a form of restricted delamination and they acted as delamination barriers around the regions. Therefore, stitched/nano *p*-aramid/phenolic composites could be considered as damage tolerant materials.

## Abstract 2

The bending properties of four types of nanostitched and nanoprepreg three dimensional (3D) *para*-aramid/phenolic composite were studied. Stitched or stitched/nano *p*-aramid/phenolic composites could be considered as damage tolerant materials.

## Graphical abstract

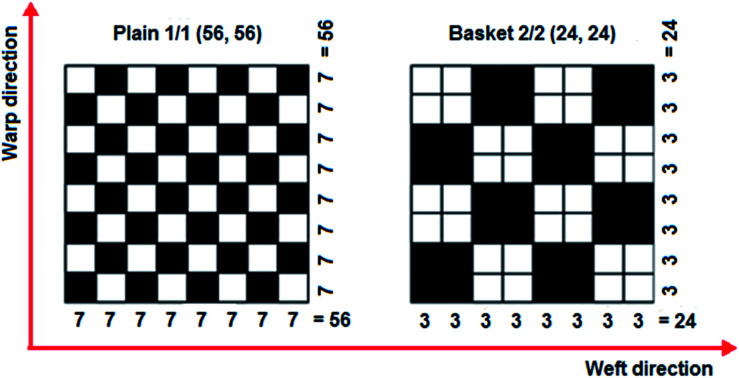

## 1. Introduction

Fiber based materials are employed in the space and aerospace industries due to their high thermo-mechanical and damage tolerance properties.^1–3^ However, they suffer from delamination. In order to develop a delamination-free structure, *Z*-directional preforms were developed by three dimensional (3D) weaving,^4^ 3D braiding,^5^ and stitching techniques.^6–8^ Recently, nanospheres, or single-wall or multiwall tubes have been employed in fiber composites by dispersing the nanomaterials in resin.^9^ If nanofibers are used, they are attached, grown or grafted onto one dimensional fibers or two dimensional (2D) fabrics.^10^ 3D composites are considered to have low plane properties due to *Z*-fibers in the thickness. Nanospheres, nanotubes or nanofibers are all randomly distributed in the fabric and they do not provide true out-of-plane reinforcement to the structure due to their discontinuous forms. The number of fabric directional fiber ends per cm and the amount of crimp affect the flexural rigidity of the dry fabric. Multistitched layered preforms show high bending rigidity due to stitching. Inter-layer deformations to the thickness direction decrease and are not easily formed.^11^

The tensile and flexural properties of the 2D fabric composites were improved by stitching because of inter-layer stress distribution.^12,13^ It was demonstrated that crack propagation in the composite was suppressed by the increase of stitching density.^14^ However, one of the experimental studies showed that the flexural strength of a stitched *E*-glass composite decreased due to stress concentration.^15,16^ The flexural strength in the unstitched *E*-glass/polyester composite was influenced by the yarn orientation, composite fiber volume fraction and preform packing density. It showed mode-I delamination as a form of inter-layer opening.^17^ A fiber distortion model was proposed to overcome the heterogeneous fiber volume fraction throughout the composite due to stitching, which affected fiber misalignment during the stitching process.^18^ Stitching the fibers did not affect the stiffness of the stitched composite.^19^ However, stitching caused weak resin-rich regions near the stitching loop section and affected the damage initiation force.^20–22^ It was claimed that the flexural strength and modulus of the 3D multiaxis composite were hardly lower than those of the 3D composite because of the bias fibers.^23,24^

In single-wall carbon nanotubes (SWCNTs) and multiwall carbon nanotubes (MWCNTs), sampling, size, surface area/volume, density, crystallinity and purity are considered important material parameters.^25–30^ The modulus of the nanocomposite decreases because of agglomeration during the consolidation process.^31^ Therefore, MWCNTs were functionalized by silanization to prevent early stage clustering and it was proven that functionalization improved the homogeneous dispersion of the nanoparticles.^32,33^ Another study showed that the thermo-mechanical properties of the nanocomposite were enhanced by grafting silane in the CNTs due to the improved inter-layer bonding and even dispersion of the nanotubes.^34,35^ Amine coated SWCNTs improved the fatigue properties of the carbon/epoxy composite.^36^ In addition, the inter-layer properties of the *E*-glass composite increased because of the coated nanotubes.^37^ A carboxyl-functionalized MWCNT (0.1–0.4%)/epoxy nanocomposite was made and it was demonstrated that its flexural properties were improved compared to the epoxy composite.^38^ The multiscale composites had improved flexural properties compared to the neat composites. This was because of a better interface between the amino coated nanoparticles and the resin, which enhanced the load transfer mechanisms. However, the presence of minor clustering adversely influenced the load-carrying mechanism.^39^ It was claimed that a naphthalene diimide and poly(dimethylsiloxane) based dispersant was synthesized to enhance the agglomeration of the SWCNTs in the matrix.^40^ It was reported that the bending modulus of the binary nanocomposite showed an improvement of about one-third compared to the neat composite.^41^ In addition, the carbon fiber surface characteristics were also found to have a significant effect on the bending properties of the composite.^42^ It was mentioned that nano-silicon carbide affected the material’s modulus but its homogeneous dispersion with the coupling agent influenced the material’s strength.^43^ However, the tensile strength of the *E*-glass composite declined by increasing the amount of nano-silicon carbide because of the interface characteristics of the nano-resin region, which caused stress concentration.^44^ Another study showed that the tensile strength modulus of *E*-glass/polyester was improved with an increase of nanosilica.^45,46^ Also, multistitching and nanosilica in the *E*-glass composite led to improved damage resistance.^45,46^ Multiwall carbon nanofibers (MWCNFs) were vertically grown on the fiber or fabric surface by chemical deposition using ethyne (C_2_H_2_) and an iron dichloride catalyst (FeCl_2_).^47^ Spun yarn of MWCNFs (1 mm length, 50 nm diameter) was drawn from an MWCNT array by the dry-spinning technique.^48^ The MWCNF spun yarn was pultruded as a 7-ply cord/epoxy rod. It was noted that the spun nanocarbon fiber based pultruded epoxy rod had better tensile strength and modulus compared to the base epoxy, and the dominant failure mode was nanofiber breakage.^49^ Hollow halloysite nanotubes (HNT, nanoclays) were employed as nanocontainers for protection of the cellulosic materials.^50^ In addition, a natural wax/HNT nanocomposite was introduced to repair the archaeological cellulosic materials.^51^ It was also claimed that a renewable polymer/HNTs composite film was made for barrier and delivery applications.^52^

A few studies were carried out on the nano-added stitched structures. The research concentrated on the flexural properties of the *p*-aramid/phenolic composite, which was developed by nanoparticles and multistitching. The objective of this study was to develop nanostitched and nanoprepreg *p*-aramid/phenolic carbon nanotube (MWCNT) composites and to examine the flexural properties of these structures.

## 2. Materials and methods

### 2.1 3D *p*-aramid/phenolic nanopreform and nanocomposite

**Fig. 1:**
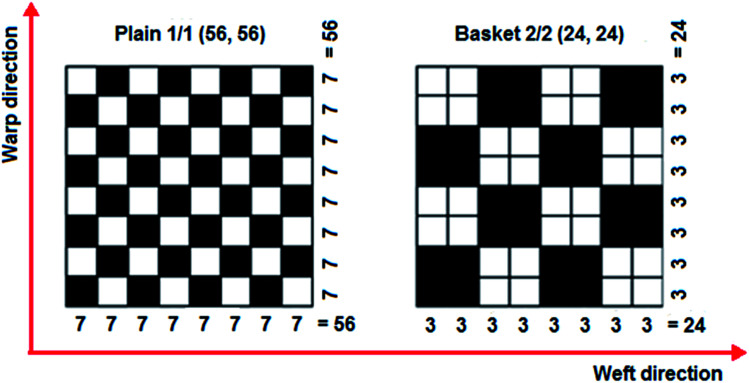
Schematic views of interlacement placement in the *p*-aramid fabrics (plain 1/1 and basket 2/2 weaves) and number of yarn interlacements in each fabric direction.

*Para*-aramid Twaron® plain (1/1) fabric (CT 747, Teijin, JP) and basket (2/2) fabric (CT736, Teijin, JP) were employed to make the multi-stitched 3D nanopreform. The *p*-aramid fabric specifications are provided in Table 1. The *p*-aramid fabric was formed from 336 tex fiber for the plain (1/1) weave and 168 tex fiber for the basket (2/2) weave. The warp and filling densities of the plain (1/1) and basket (2/2) *p*-aramid fabrics were 62.5 ends per 10 cm and 127 ends per 10 cm, respectively. The *p*-aramid fabric unit area weights and thickness were 410 g m^−2^ and 0.62 mm for both fabrics, respectively. The directional interlacements of plain (1/1) and basket (2/2) are schematically represented in Fig. 1. The number of interlacements of the plain and basket fabrics were 56 and 24, respectively, and their placements in the fabrics were homogeneously distributed. The multiwall carbon nanotubes (MWCNTs, Nanothinx, GR) were selected based on better thermo-mechanical properties and commercial availability. The average size of the MWCNTs varied from 15–35 nm in diameter, 10 μm in length and 1–2 nm for wall thickness. The tensile strength and modulus of the MWCNTs were 200 GPa and 1 TPa, respectively, as presented in Table 2.

**Table 1:** Specifications of the *p*-aramid fabric structures

Fabric label	Weave type	Fabric treatment	Yarn linear density (tex)		Fabric density (per 10 cm)		Fabric area weight (g m^−2^)	Yarn crimp (%)		Fabric thickness (mm)
			Warp	Weft	Warp	Weft		Warp	Weft	
Twaron CT® 747	Plain (1/1)	Water repellent	336	336	62.50	62.50	410	5.80	5.90	0.62
Twaron CT® 736	Basket (2/2)	Water repellent	168	168	127	127	410	9.40	11.30	0.62

**Table 2:** Specifications of the multiwall carbon nanotubes (MWCNTs)^25,53^

Nanomaterial	Particle dimensions (diameter × length × wall thickness) (nm × micron × nm)	Surface area (m^2^ g^−1^)	Purity (%)	Density (g cm^−3^)	Tensile strength (GPa)	Tensile modulus (TPa)	Melting temperature (°C)
Carbon nanotubes (MWCNTs, Nanothinx, GR)	15–35 × 10≥ × 1–2≥	>100	≥97	1.74	200	1.0	3550

**Fig. 2:**
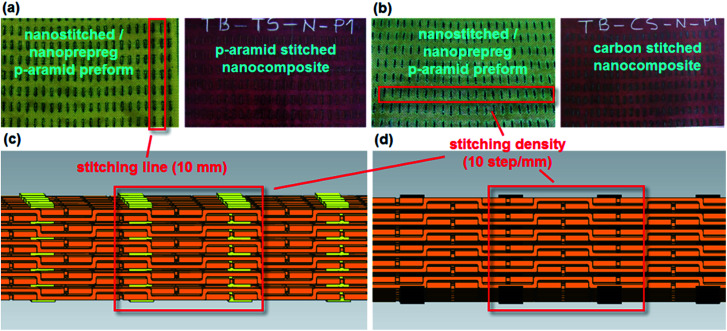
(a) *Para*-aramid Twaron CT multistitched 3D nanoprepreg preform (left) and *p*-aramid/phenolic MWCNT composite (right) (TB–TS–N); (b) PAN carbon multistitched 3D nanoprepreg preform (left) and *p*-aramid/phenolic MWCNT composite (right) (TB–CS–N); (c) schematic view of *p*-aramid stitched *p*-aramid structure (TB–TS); (d) schematic view of carbon stitched *p*-aramid structure (TB–CS).

Principally, four types of *p*-aramid structure were developed. These were base (TPU, TBU), in which TPU was a six-layer [(0°/90°)]_6_*p*-aramid plain (1/1) woven fabric, while TBU was a six-layer *p*-aramid basket (2/2) woven fabric; stitched (TP–CS, TP–TS, TB–CS, TB–TS), in which TP–CS and TP–TS were six-layer *p*-aramid plain (1/1) woven fabric with one-directionally PAN carbon and *p*-aramid Twaron CT stitched in warp (0°), respectively, whereas TB–CS and TB–TS were six-layer basket (2/2) woven fabric with one-directional carbon and Twaron CT stitched in warp (0°) structures, respectively; base/nano (TPU–N, TBU–N), in which TPU–N and TBU–N were six-layer *p*-aramid plain (1/1) and basket (2/2) woven fabric, respectively, with added MWCNTs; and stitched/nano (TP–CS–N, TP–TS–N, TB–CS–N, TB–TS–N). When the MWCNTs were added to all the stitched structures described above, they were considered as stitched/nano structures. One-directional stitching was manually made on the layered woven structures using the PAN carbon and *p*-aramid Twaron CT stitching yarns, as shown in Fig. 2 (a and b). The stitching fiber properties are also provided in Table 3.

**Table 3:** Specifications of untwisted stitching yarns^a^

Fiber type	Fiber diameter (μm)	Fiber density (g cm^−3^)	Tensile strength (GPa)	Tensile modulus (GPa)	Elongation at break (%)	Yarn linear density (dtex)
Twaron CT (*para*-aramid fiber, Teijin, JP)	12	1.45	3.2	115	2.9	3360
Polyacrylonitrile (PAN) carbon (carbon fiber, Aksaca, TR)	6	1.78	4.2	240	1.8	6 K^1^

a K^1^: 1000 filaments in the untwisted fiber TOW.

**Fig. 3:**
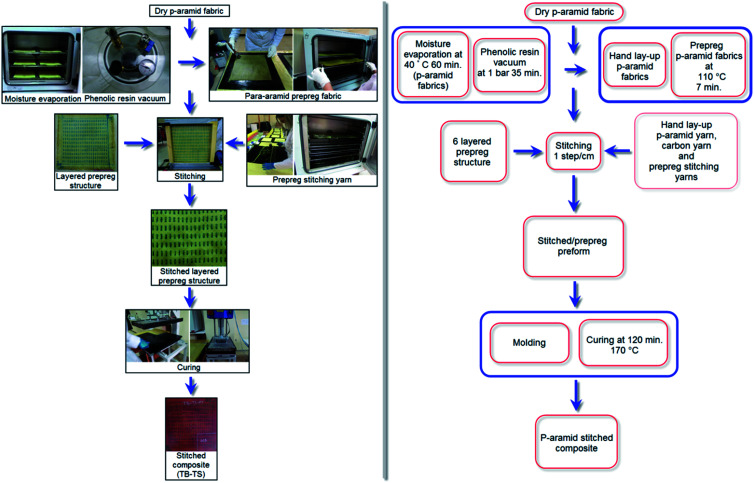
Processing steps of one-directional stitched multilayered *p*-aramid/phenolic woven prepreg preforms and composites (TB–TS).

**Fig. 4:**
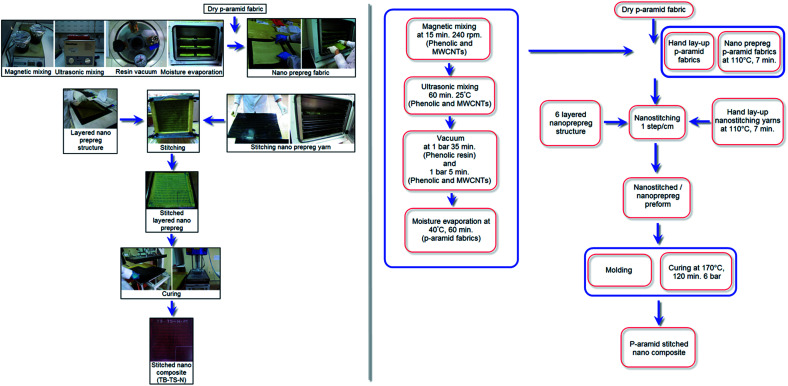
Processing steps of one-directional stitched multilayered *p*-aramid/phenolic/carbon nanotube woven prepreg preforms and composites (TB–TS–N).

Stitched/nano multilayer *p*-aramid woven preforms were consolidated to make stitched/nano *p*-aramid composites. Fig. 3 and 4 show the processing steps for the one-directional stitched *p*-aramid/phenolic and stitched/nano *p*-aramid/phenolic composites, respectively. Initially, phenolic resin (Araldite EPN 1138, Biesterfeld Spezialchemie GmbH, DE) was put into a vacuum chamber (Metyx composites, TR) under 0.1 MPa pressure (1 bar) for 35 minutes to remove any air bubbles. Then, MWCNTs (0.03125, %wt) were added to the phenolic resin. In order to conduct pre-mixing to prevent possible heterogeneous dispersion and early agglomeration, they were stirred by a magnetic mixer (Wisestir®, Witeg, DE) at 240 rpm for 15 minutes. Immediately afterward, the phenolic/carbon nanotube solution was mixed in an ultrasonic bath (200 watt, 40 kHz, DAIHAN/WiseClean®, WUC-A03H, KR) at 25 °C for 60 minutes. Therefore, a highly homogenized phenolic/nano mixture was obtained. Again, this was stirred by a magnetic mixer at 240 rpm for about 15 minutes to improve the homogenization and agglomeration of the mixture. Then it was placed into a vacuum chamber again under 0.1 MPa pressure for 5 minutes to remove the remaining air bubbles. At the same time, plain (1/1) and basket (2/2) *p*-aramid woven fabrics, polyacrylonitrile (PAN) carbon fibers (6 K, Aksaca, TR) and *para*-aramid Twaron CT (3360 dtex, Teijin, JP) yarn were prepared to make the flexure test plates. We first of all made the *p*-aramid/phenolic carbon nanotube prepreg fabrics and yarns, and then they were consolidated for the composites.

The *para*-aramid fabric was heated at 40 °C for 60 minutes to evaporate the moisture. Next, the matrix was applied to the *p*-aramid fabric by the hand layup method under atmospheric conditions. It was put on the shelf of an oven (Binder, DE) to pre-cure at 110 °C for 7 minutes in order to obtain the prepreg nano *para*-aramid fabric. The same procedure was applied to the carbon and *para*-aramid stitching yarns to make the prepreg yarns. The prepreg nano *p*-aramid fabric was layered as a [0°/90°]_6_ sequence. The six-layered prepreg nano *p*-aramid preform was manually stitched by carbon or *p*-aramid nano stitching yarn using an in-house developed apparatus to make the stitched/nano composite. The density of stitching was 1 step per cm. The space between neighboring stitching lines was 1 cm. The stitched prepreg *p*-aramid/phenolic carbon nanotube preform was put in a mold, and the mold was wrapped with Teflon film (FDM 2100, DuPont, USA) to prevent thermal shock and easy demolding after curing. The mold was cured using a hot press (Climax, TR) under 0.6 MPa pressure (6 bar) at 170 °C for 120 minutes. Lastly, the mold was left in the press to cool until the temperature was gradually decreased to 40 °C and the stitched *p*-aramid/phenolic carbon nanotube composite was removed from the mold. Some of the composites are shown in Fig. 2 (a and b).

The densities of the stitched/nano carbon composites were found by ASTM D792-91.^54^ It was designed to find the density (g cm^−3^) as the sample mass in air divided by its volume, whereas the relative density was the sample density divided by the density of water. The composite volume fraction and void content were obtained by ASTM D3171-99 (ref. 55) and ASTM D2734-91,^56^ respectively. In the determination of the composite fiber volume fraction, once the sample mass and density were known, the furnace was heated up to 400 °C. Then, the composite sample was kept inside for almost 5.5 hours to remove the burned matrix. The remaining residue, which contained the *p*-aramid fiber in the fabric, was then cooled and weighed. The weight percent of the fiber in the composite was then calculated. In addition, the void content was also calculated from known parameters such as the matrix and composite densities. After the flexure test, the delaminated areas and damaged surfaces of the composite sample were analyzed by an optical microscope (Olympus SZ61, JP equipped with Bs200DOC digital image analysis software-Bs200DOC, TR).

### 2.2 Flexural test

**Fig. 5:**
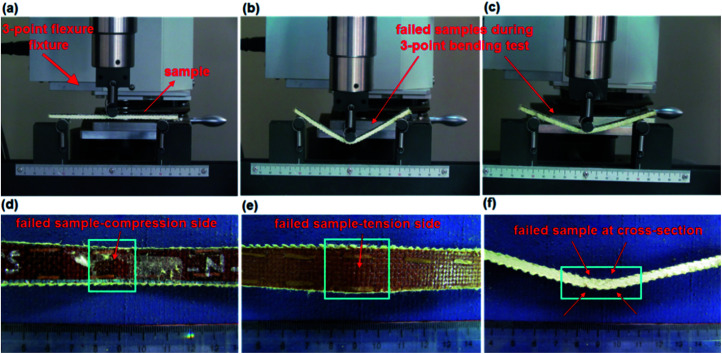
(a) Tensile tester with flexural fixture with a sample at the initial state; (b) base/nano sample during the application of the bending load (TBU–N); (c) stitched/nano sample during the application of the bending load (TB–TS–N); (d) compression side of the failed stitched/nano sample (TB–TS–N); (e) tension side of the failed stitched/nano sample (TB–TS–N); (f) failed stitched/nano sample at cross-sections (TB–TS–N, digital image).

The three-point flexural tests of all the composites were carried out on a Shimadzu AG-XD 50 (Japan) tester equipped with Trapezium® software with a 5 kN loading cell based on ASTM D790-10.^57^ The bending testing speed was 1.3653 mm min^−1^. The test dimensions were 12.7 (width) × 130 mm (length). The *L*/*d* (support span length/thickness) ratio was 32/1. The flexural load applied to each sample was the warp (0°, lengthwise). Fig. 5 shows the bending test instrument and fixture with samples. Eqn (1)–(3) present the flexural strength, modulus and strain, respectively.^57^ The flexural test was conducted under the standard laboratory atmosphere with a temperature of 23 °C ± 2 °C and relative humidity of 50% ± 10%. After the bending load was applied to the samples, they were examined by an optical microscope (Olympus SZ61, Japan).1
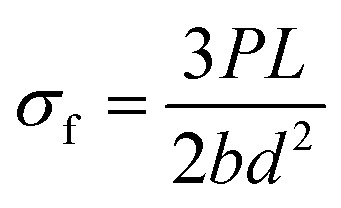
2
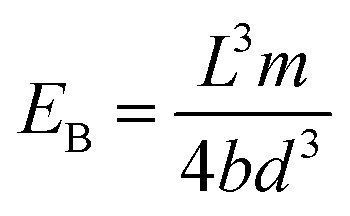
3
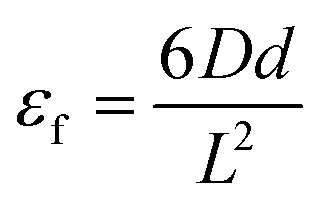
where *σ*_f_ is the flexural strength in the outer fibers at midspan (MPa); *P* is the load at a given point on the load-deflection curve (N); *L* is the support span (mm); *b* is the width of the beam tested (mm); *d* is the depth of the beam tested (mm); *E*_B_ is the modulus of elasticity in bending (GPa); *m* is the slope of the tangent to the initial straight-line portion of the load-deflection curve; *ε*_f_ is the bending strain (%); *D* is the maximum displacement of the center of the beam (mm).

## 3. Results and discussion

### 3.1 Density and fiber volume fraction results

The density and fiber volume fraction results of base (TPU, TBU), stitched (TP–CS, TP–TS, TB–CS, TB–TS), base/nano (TPU–N, TBU–N) and stitched/nano (TP–CS–N, TP–TS–N, TB–CS–N, TB–TS–N) composites were evaluated. The densities of the developed structures varied from 1.30–1.33 g cm^−3^ and the average density was 1.32 g cm^−3^. The density differences in the structures were considered to be negligible (1%). The measured total fiber weight fractions (*V*_tfw_) of all structures varied from 67.10–73.81% and the average total fiber weight fraction was 69.84%. The volume fraction differences between the structures were around 4–6% due to the stitching yarn weight fraction and MWCNT addition as well as a minor stitching effect on the preforms. The measured stitching fiber weight fractions (*V*_sfw_) of all the stitched and stitched/nano structures varied from 1.47–2.13% and the average stitching fiber weight fraction was 1.81%. The void content (weight base, *V*_c_) of all the structures varied from 0.71–1.83% (average 1.26%). These results were obtained assuming that all the structures were made under defect-free processing conditions from preform preparation to consolidation.

**Fig. 6:**
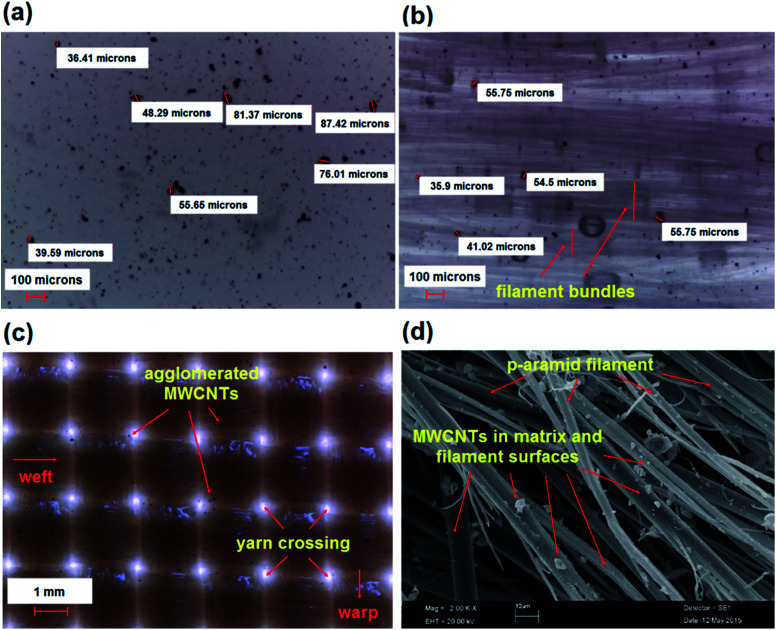
(a) MWCNT dispersion in phenolic resin; (b) phenolic/MWCNT treated uncured nanoyarn; (c) phenolic/MWCNT treated uncured nanofabric surface; (d) fractured nanostitched yarn in stitched/nano composite (TB–TS–N) (optical photos, magnification ×40, ×40 and ×10, respectively; SEM, magnification ×2000).^58^

On the other hand, the addition and dispersion of MWCNTs in the phenolic resin was analyzed during processing. We started by selecting a 0.5% (weight%) ratio for the MWCNTs as an initial condition. Afterward, a large agglomeration (about 200–300 microns) of nanotubes was found in the phenolic resin. Extensive studies were conducted to decrease the extent of agglomeration of the nanotubes. For this reason, we decreased the MNCNT ratio to 0.03125% and increased the stirring time from 60 minutes to 120 minutes in ultrasonic mixing. Therefore, the size of the agglomeration of carbon nanotubes decreased to 30–80 microns in the phenolic resin, as shown in Fig. 6 (a). The phenolic resin with added MWCNTs was applied to the sample stitching yarn and fabric. The MWCNTs were evenly dispersed in the filament direction and intra-filament regions of the stitching yarn and in the principle directions and yarn crossing regions of the fabric, as shown in Fig. 6 (b) and (c), respectively. Fig. 6 (d) shows the MWCNT distribution in the filaments of the fractured nanostitched yarn in the stitched/nano composite.

### 3.2 Flexural results

**Fig. 7:**
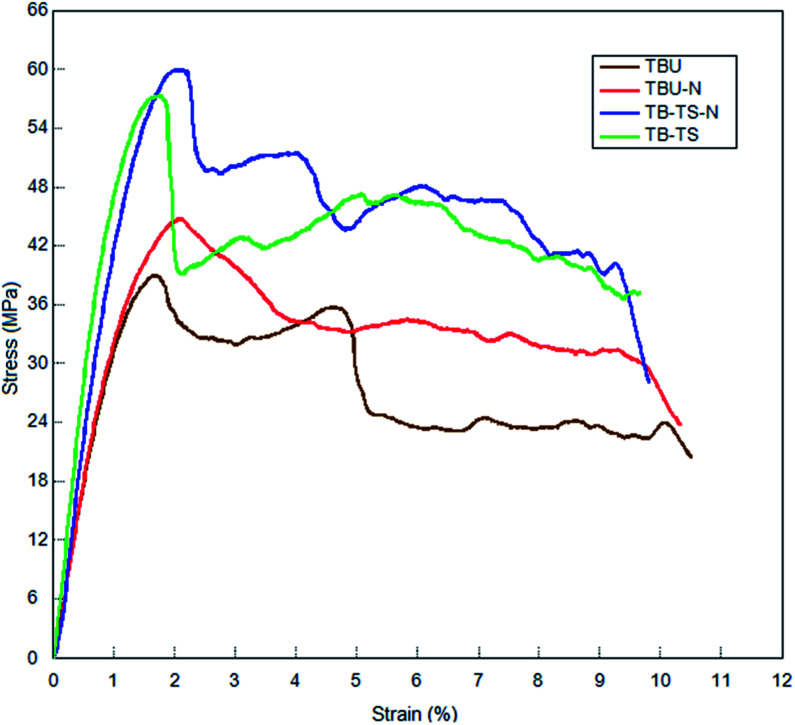
Stress–strain curves from the flexure test for some of the multistitched carbon/epoxy MWCNT composites (base TBU; base/nano TBU–N; stitched TB–TS; stitched/nano TB–TS–N).

The flexure test results of the base (TPU, TBU), stitched (TP–CS, TP–TS, TB–CS, TB–TS), base/nano (TPU–N, TBU–N) and stitched/nano (TP–CS–N, TP–TS–N, TB–CS–N, TB–TS–N) composites are given in Table 4. The data presented in Table 4 are the average values of flexure strength, strain and modulus for each composite. Although we claimed that all the structures were produced without defects, they probably contain microscopic nonlinearities at the stitching piercing region of the nanoprepreg preforms, especially in the out-of-plane direction, heterogeneous distributions of the MWCNTs in the preform surface and intra-layer sections, and minor agglomerations of the MWCNTs in the matrix and fabric interlacement regions. Therefore, these partly affect the ability to obtain reproducible data from the flexural tests. The flexural test results in Table 4 also include the standard deviation (*s*) and the coefficient of variation (CV%), where the CVs of the flexural strength and modulus varied from 1.77–9.41% and 1.80–17.12%, respectively. Fig. 7 shows the tensile stress–strain curves of some of the basket (2/2) fabric based composites. In Fig. 7, the stress–strain curve of the basket *p*-aramid/phenolic structure is presented together with those of its base, nano, carbon and *para*-aramid stitched, and stitched/nano forms. The *p*-aramid stitched and stitched/nano structures showed higher flexure strength values compared to the base and base/nano structures. In addition, the stress–strain curves almost perfectly became the same line, beginning at the initial state in the elastic region to the failure points, at which there were no sharp drops.

**Table 4:** Average flexural test results of various developed *p*-aramid/phenolic MWCNT composites^a^

Label	Flexural load (max.) (N)	Flexural displacement (mm)	Flexural strain (%)	Flexural strength (MPa)	Flexural modulus (GPa)
TPU	64.43	8.73	2.09	40.51, (*s* = 1.98, CV% = 4.89)	3.64, (*s* = 0.07, CV% = 1.80)
TBU	57.90	7.81	1.84	39.01, (*s* = 0.69, CV% = 1.77)	3.44, (*s* = 0.18, CV% = 5.11)
TP–CS	56.18	6.81	1.59	38.91, (*s* = 3.28, CV% = 8.42)	4.21, (*s* = 0.36, CV% = 8.58)
TP–TS	53.87	7.73	1.75	42.78, (*s* = 3.15, CV% = 7.35)	4.16, (*s* = 0.71, CV% = 17.12)
TB–CS	79.59	9.04	2.01	57.24, (*s* = 3.77, CV% = 6.58)	4.90, (*s* = 0.44, CV% = 8.92)
TB–TS	61.57	8.36	1.80	57.73, (*s* = 2.53, CV% = 4.38)	5.25, (*s* = 0.83, CV% = 15.89)
TPU–N	64.11	9.43	2.13	46.12, (*s* = 1.62, CV% = 3.51)	3.86, (*s* = 0.34, CV% = 8.70)
TBU–N	61.69	9.92	2.23	45.37, (*s* = 2.83, CV% = 6.24)	3.72, (*s* = 0.11, CV% = 2.98)
TP–CS–N	56.59	7.78	1.78	40.59, (*s* = 3.82, CV% = 9.41)	3.79, (*s* = 0.15, CV% = 3.95)
TP–TS–N	55.65	8.44	2.03	35.45, (*s* = 3.11, CV% = 8.76)	2.85, (*s* = 0.42, CV% = 14.69)
TB–CS–N	66.40	8.28	1.94	45.30, (*s* = 2.82, CV% = 6.21)	3.65, (*s* = 0.52, CV% = 14.35)
TB–TS–N	75.54	9.29	2.02	60.45, (*s* = 2.78, CV% = 4.60)	4.87, (*s* = 0.38, CV% = 7.85)

a “*s*” represents the standard deviation and “CV%” represents the coefficient of variation.

### 3.3 Flexural strength

**Fig. 8:**
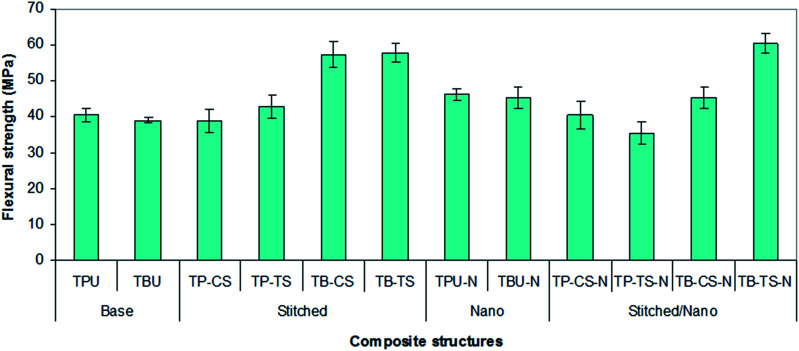
Flexure strength of various developed *p*-aramid/phenolic MWCNT composites.

Fig. 8 shows the average flexure strength values of all the developed *p*-aramid/phenolic MWCNT composites. As shown in Fig. 8 and Table 4, the flexure strength of the base (TBU and TPU) composites varied between 39.01–40.51 MPa, whereas the flexure strength of the base/nano (TBU–N and TPU–N) composites varied between 45.37–46.12 MPa. The flexure strength of the stitched (TP–CS, TP–TS, TB–CS and TB–TS) composites varied between 38.91–57.73 MPa, whereas the flexure strength of the stitched/nano (TP–CS–N, TP–TS–N, TB–CS–N and TB–TS–N) composites varied between 35.45–60.45 MPa. The bending strength of the *p*-aramid stitched/nano basket 2/2 (TB–TS–N) composite was 4.50% higher for the stitched (TB–TS) and 35.47% for the base (TBU), whereas the flexure strength of the PAN carbon stitched/nano basket 2/2 (TB–CS–N) was 20.86% lower for the stitched (TB–CS) and 13.89% higher for the base (TBU). The *p*-aramid nanostitched composite (TB–TS–N) showed better performance (25.06%) compared to the PAN carbon nanostitched composite (CT–CS–N), whereas the *p*-aramid stitched structure (CT–TS) demonstrated very slightly better performance (0.90%) compared to the stitched (CT–CS) composite. In addition, the flexure strength of the base/nano (TBU–N) was 14.02% higher than that of the base (TBU). It was realized that stitching and MWCNTs slightly increased the bending strength of all the stitched and stitched/nano composites. The stitching fiber type also slightly affected the flexural strength of the stitched and stitched/nano composites. We also obtained similar results for the *p*-aramid plain 1/1 composites.

### 3.4 Flexural strain

**Fig. 9:**
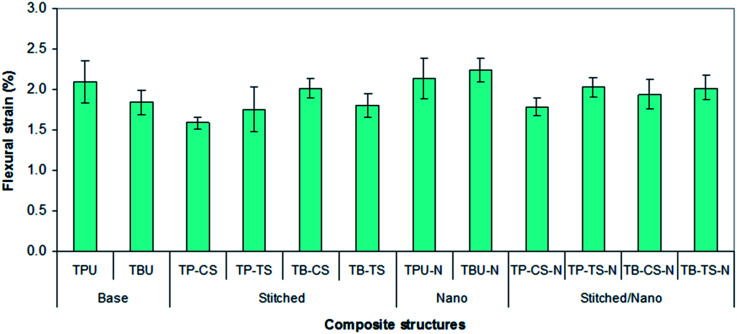
Flexure strain of various developed *p*-aramid/phenolic MWCNT composites.

Fig. 9 shows the average flexure strain of all the developed *p*-aramid/phenolic MWCNT structures. In Fig. 9 and Table 4, the flexure strain of the base (TBU and TPU) composites varied between 1.84–2.09%, whereas the flexure strain of the base/nano (TBU–N and TPU–N) composites varied between 2.13–2.23%. The flexure strain of the stitched (TP–CS, TP–TS, TB–CS and TB–TS) composites varied between 1.59–2.01%, whereas the flexure strain of the stitched/nano (TP–CS–N, TP–TS–N, TB–CS–N and TB–TS–N) composites varied between 1.78–2.03%. The flexure strain of the *p*-aramid stitched/nano basket 2/2 (TB–TS–N) composite was 10.90% higher for the stitched (TB–TS) and 8.91% higher for the base (TBU), whereas the flexure strain of the PAN carbon stitched/nano basket 2/2 (TB–CS–N) was 3.48% lower for the stitched (TB–CS) and 5.15% higher for the base (TBU). The *p*-aramid nanostitched structure (TB–TS–N) showed better performance (3.96%) compared to the PAN carbon nanostitched composite (TB–CS–N), whereas the *p*-aramid stitched structure (TB–CS) demonstrated better performance (10.44%) compared to the stitched (TB–TS). In addition, the flexure strain of the base/nano (TBU–N) was 17.49% higher than that of the base (TBU). It was realized that stitching and MWCNTs hardly increased the bending strain of all the stitched and stitched/nano composites. The stitching fiber type also slightly affected the bending strain of the stitched and stitched/nano composites. On the other hand, we did not obtain consistent results for all the *p*-aramid plain 1/1 composites.

### 3.5 Flexural modulus

**Fig. 10:**
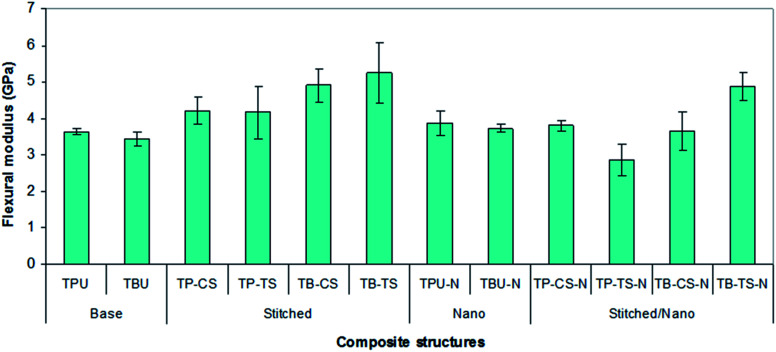
Flexure modulus of various developed *p*-aramid/phenolic MWCNT composites.

Fig. 10 shows the average flexure modulus values of all the developed *p*-aramid/phenolic MWCNT structures. In Fig. 10 and Table 4, the flexure modulus of the base (TBU and TPU) composites varied between 3.44–3.64 GPa, whereas the flexure modulus of the base/nano (TBU–N and TPU–N) composites varied between 3.72–3.86 GPa. The flexure modulus of the stitched (TP–CS, TP–TS, TB–CS and TB–TS) composites varied between 4.16–5.25 GPa, whereas the flexure modulus of the stitched/nano (TP–CS–N, TP–TS–N, TB–CS–N and TB–TS–N) composites varied between 2.85–4.87 GPa. The flexure modulus of the *p*-aramid stitched/nano basket 2/2 (TB–TS–N) composite was slightly (7.24%) lower for the stitched (TB–TS) and 29.36% higher for the base (TBU), whereas the flexure modulus of the PAN carbon stitched/nano basket 2/2 (TB–CS–N) was 25.51% lower for the stitched (TB–CS) and 5.75% higher for the base (TBU). The *p*-aramid nanostitched structure (TB–TS–N) showed better performance (25.05%) compared to the PAN carbon nanostitched composite (CT–CS–N), whereas the *p*-aramid stitched structure (TB–TS) demonstrated slightly better performance (6.67%) compared to the stitched (TB–CS) composite. In addition, the flexure modulus of the base/nano (TBU–N) was 7.53% higher than that of the base (TBU). It was found that stitching and MWCNTs slightly affected the bending modulus of all the stitched and stitched/nano composites. The bending modulus was also somewhat affected by the stitching yarn type. However, we did not obtain consistent results for all the *p*-aramid plain 1/1 structures.

### 3.6 Failure after flexural test results

**Fig. 11:**
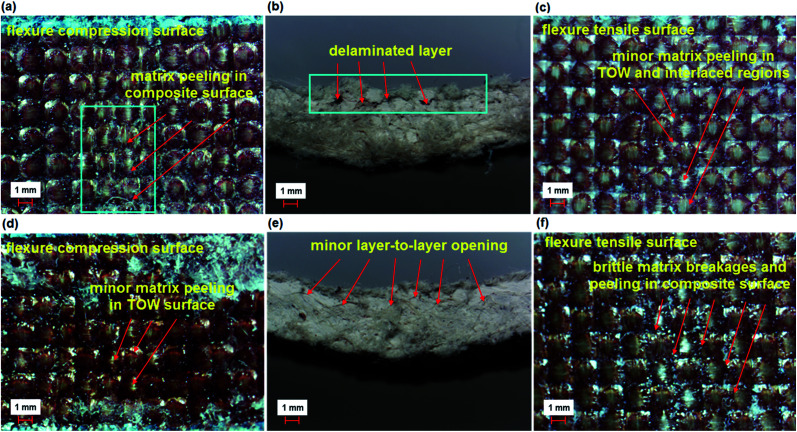
Warp directional flexure failure in various multistitched 3D *p*-aramid/phenolic MWCNT composites. (a) Base top face (TBU); (b) base cross-section (TBU); (c) base bottom face (TBU); (d) base/nano top face (TBU–N); (e) base/nano cross-section (TBU–N) and (f) base/nano bottom face (TBU–N) (optical microscope, magnification ×6.7).

**Fig. 12:**
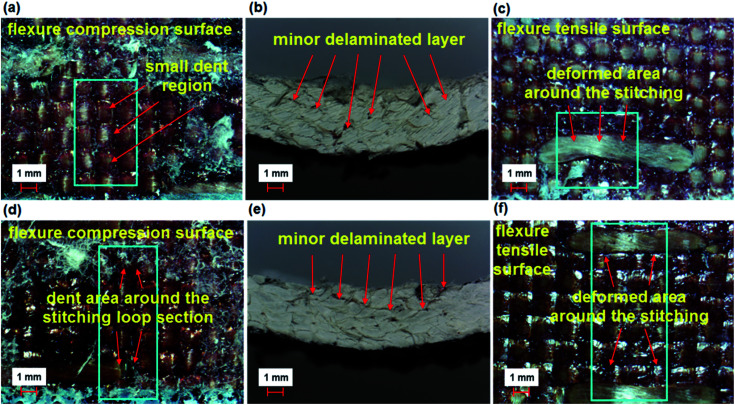
Warp directional flexure failure in various multistitched 3D *p*-aramid/phenolic MWCNT composites. (a) Stitched top face (TB–TS); (b) stitched cross-section (TB–TS); (c) stitched bottom face (TB–TS); (d) stitched/nano top face (TB–TS–N); (e) stitched/nano cross-section (TB–TS–N) and (f) stitched/nano bottom face (TB–TS–N) (optical microscope, magnification ×6.7).

**Fig. 13:**
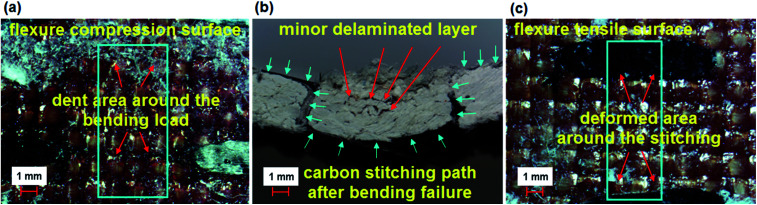
Warp directional flexure failure in PAN carbon nanostitched *p*-aramid/phenolic MWCNT composites. (a) Stitched/nano top face (TB–CS–N); (b) stitched/nano cross-section (TB–CS–N); (c) stitched/nano bottom face (TB–CS–N) (optical microscope, magnification ×6.7).

The flexure failures of some base (TBU), base/nano (TBU–N), stitched (TB–TS), and stitched/nano (TB–TS–N and TB–CS–N) composites are presented in Fig. 11–13. The damaged areas created by the bending load for each sample were barely visible. Therefore, we did not measure the damaged areas. Some of the bending failures of the base (TBU) and base/nano (TBU–N) composites are shown in Fig. 11 (a–f). The tension side of the base (TBU) and base/nano (TBU–N) structures had outwardly lateral multiple warp directional bending and local matrix peeling, and no visible fiber breakages were found (Fig. 11 (c and f)). The compression side of the base (TBU) and base/nano (TBU–N) structure had inwardly warp directional bending and lateral matrix peeling, and no visible fiber failures were obtained (Fig. 11 (a and d)). In the cross-section of the TBU, a delaminated layer near the top surface was observed, whereas various local angular delaminated areas were found near to the mid-surface line (Fig. 11 (b and e)).

Some of the bending failures of the stitched (TB–TS) and stitched/nano (TB–TS–N) composites are shown in Fig. 12 (a–f). The tension side of the stitched (TB–TS) and stitched/nano (TB–TS–N) structures had outwardly lateral deformation on warp and no visible matrix/fiber damages were found (Fig. 12 (c and f)). The compression side of the stitched (TB–TS) and stitched/nano (TB–TS–N) structures had inwardly dented areas around the stitching lines and no matrix/fiber damages were identified (Fig. 12 (a and d)). In the cross-section of the TB–TS, minor delaminated layers were observed around the mid-plane of the structure. Some local angular delaminated areas between the mid-plane line and the top surface were found (Fig. 12 (b and e)). The results of bending failure showed that all the developed *p*-aramid/phenolic structures were flexible and did not suffer brittle fiber breakages. The addition of stitching and MWCNTs to the base structures made them delamination-restricted materials.

One of the bending failures of the PAN carbon stitched/nano (TB–CS–N) composite is shown in Fig. 13 (a–c). The tension side of the stitched/nano structure had a deformed area around the strained stitching line (Fig. 13 (c)). However, the compression side of the TB–CS–N had a dented area around the stitching step (Fig. 13 (a)). In the cross-section, the minor delaminated area was restricted by stitching, where the stitched fiber acted as a delamination barrier around the region (Fig. 13 (b)).

## 4. Conclusions

Stitched/nano *p*-aramid/phenolic composites were developed and their bending properties were studied. The flexure failure of the developed composites was also analyzed. The addition of stitching and multiwall carbon nanotubes to the base structures slightly increased the flexure strength, modulus and strain of all the stitched and stitched/nano composites. However, we did not generally obtain consistent results in all the stitched, nano and stitched/nano composites, in particular the plain 1/1 pattern fabric composites. It was also found that the type of stitching fiber slightly affected the flexural properties of the *p*-aramid/phenolic composites.

The flexure failure in the tension side of the base and base/nano structures was matrix peeling and no visible fiber breakages and large delaminated areas were found near the top surface and mid-plane line, whereas the stitched and stitched/nano composites had warp deformation but no visible matrix/fiber damage and the delaminated areas were severely restricted and small crack propagation was obtained. The flexure failure in the compression side of the base and base/nano structures was lateral warp bending; lateral matrix peeling but almost no visible fiber damage was observed, whereas the stitched and stitched/nano composites had lateral small dented regions and no visible matrix/fiber breakages. The results showed that the addition of the stitching fibers and multiwall carbon nanotubes in the base structure improved the out-of-plane failure properties by restricting delamination and they acted as delamination barriers around the regions. Stitched or stitched/nano *p*-aramid/phenolic composites could be considered as damage tolerant materials.

## Funding

This research received a grant from Roketsan Industries Incorporations Grant No. RS/ERCİYES DSM-76301-14-01N/R.

## Conflicts of interest

There are no conflicts to declare.

## Supplementary Material

## References

[cit1] Kamiya R., Cheeseman B. A., Popper P., Chou T. W. (2000). Compos. Sci. Technol..

[cit2] Chou T. W. , Microstructural Design of Fibre Composites, Cambridge University Press, New York, 1992, p. 285

[cit3] Cox B. N., Dadkhah M. S., Morris W. L., Flintoff J. G. (1994). Acta Mater..

[cit4] Bilisik K. (2012). Text. Res. J..

[cit5] Bilisik K. (2013). Text. Res. J..

[cit6] Tong L. , Mouritz A. P. and Bannister M. K., 3D Fibre Reinforced Polymer Composites, Elsevier B.V., New York, 2002, p. 163

[cit7] Bilisik K., Yolacan G. (2014). J. Compos. Mater..

[cit8] Dell’Anno G., Cartié D. D., Partridge I. K., Rezai A. (2007). Composites, Part A.

[cit9] Garcia E. J., Wardle B. L., Hart A. J. (2008). Composites, Part A.

[cit10] Khan S. U., Kim J. K. (2011). Int. J. Aeronaut. Space Sci..

[cit11] Bilisik K. (2011). Text. Res. J..

[cit12] Kang T. J., Lee S. H. (1994). J. Compos. Mater..

[cit13] Dickinson L. C., Farley G. L., Hinders M. K. (1999). J. Compos. Mater..

[cit14] Tan K. T., Watanabe N., Iwahori Y. (2012). Int. J. Damage Mech..

[cit15] Mouritz A. P., Gallagher J., Goodwin A. A. (1997). Compos. Sci. Technol..

[cit16] Mouritz A. P. (1996). Composites, Part A.

[cit17] Bilisik K., Yolacan G. (2011). J. Reinf. Plast. Compos..

[cit18] Wei Y., Zhang J. (2008). Composites, Part A.

[cit19] Wu E., Wang J. (1995). J. Compos. Mater..

[cit20] Tan K. T., Watanabe N., Iwahori Y. (2011). Composites, Part B.

[cit21] Chen G., Li Z., Kou C., Gui L. (2004). J. Reinf. Plast. Compos..

[cit22] Xiaoquan C., Al-Mansour A. M., Zhengneng L., Chenghe K. (2005). J. Reinf. Plast. Compos..

[cit23] Bilisik K. (2010). J. Reinf. Plast. Compos..

[cit24] Mohamed M. H. and Bilisik A., *US Pat.*, 5465760, 1995

[cit25] Lehman J. H., Terrones M., Mansfield E., Hurst K. E., Meunier V. (2011). Carbon.

[cit26] Iijima S. (1991). Nature.

[cit27] Hussain F., Hojjati M., Okamoto M., Gorga R. E. (2006). J. Compos. Mater..

[cit28] Thostenson E. T., Ren Z. F., Chou T. W. (2001). Compos. Sci. Technol..

[cit29] Shen L., Li J. (2005). Phys. Rev. B: Condens. Matter Mater. Phys..

[cit30] Saravanan N., Rajasekar R., Mahalakshmi S., Sathishkumar T. P., Sasikumar K. S. K., Sahoo S. (2014). J. Reinf. Plast. Compos..

[cit31] Wang H. W., Zhou H. W., Peng R. D., Mishnaevsky L. (2011). Compos. Sci. Technol..

[cit32] Ma P. C., Kim J. K., Tang B. Z. (2006). Carbon.

[cit33] Fukushima T., Kosaka A., Ishimura Y., Yamamoto T., Takigawa T., Ishii N., Aida T. (2003). Science.

[cit34] Ma P. C., Kim J.-K., Tang B. Z. (2007). Compos. Sci. Technol..

[cit35] Yu B., Jiang Z., Tang X. Z., Yue C. Y., Yang J. (2014). Compos. Sci. Technol..

[cit36] Davis D. C., Wilkerson J. W., Zhu J., Hadjiev V. G. (2011). Compos. Sci. Technol..

[cit37] Zhu J., Imam A., Crane R., Lozano K., Khabashesku V. N., Barrera E. V. (2007). Compos. Sci. Technol..

[cit38] Salam M. B. A., Hosur M. V., Jahan N., Rahman M. M., Jeelani S. (2013). Polym. Polym. Compos..

[cit39] Sánchez M., Campo M., Jiménez-Suárez A., Ureña A. (2013). Composites, Part B.

[cit40] Hong L., Takagaki Y., Yoshikawa H., Nakashima N. (2016). Bull. Chem. Soc. Jpn..

[cit41] Hosur M., Mahdi T. H., Islam M. E., Jeelani S. (2017). J. Reinf. Plast. Compos..

[cit42] Brocks T., Cioffi M. O. H., Voorwald H. J. C. (2013). Appl. Surf. Sci..

[cit43] Yongand V., Hahn H. T. (2004). Nanotech.

[cit44] Patnaik A., Satapathy A., Mahapatra S. S., Dash R. R. (2009). J. Reinf. Plast. Compos..

[cit45] Bilisik K., Yolacan G. (2014). Fibers Polym..

[cit46] Bilisik K., Yolacan G. (2014). Fibers Polym..

[cit47] Inoue Y., Kakihata K., Hirono Y., Horie T., Ishida A., Mimura H. (2008). Appl. Phys. Lett..

[cit48] Ghemes A., Minami Y., Muramatsu J., Okada M., Mimura H., Inoue Y. (2012). Carbon.

[cit49] Shimamura Y., Oshima K., Tohgo K., Fujii T., Shirasu K., Yamamoto G., Hashida T., Goto K., Ogasawara T., Naito K., Nakano T., Inoue Y. (2014). Composites, Part A.

[cit50] Cavallaro G., Danilushkina A. A., Evtugyn V. G., Lazzara G., Milioto S., Parisi F., Rozhina E. V., Fakhrullin R. F. (2017). Nanomaterials.

[cit51] Cavallaro G., Lazzara G., Milioto S., Parisi F., Sparacino V. (2015). Polym. Degrad. Stab..

[cit52] Cavallaro G., Donato D. I., Lazzara G., Milioto S. (2011). J. Phys. Chem. C.

[cit53] http://www.nanothinx.com/raw-cnts-in-powder-form/, 2017, accessed 10.01.17

[cit54] ASTM D792-91, 1991

[cit55] ASTM D3171-99, 1999

[cit56] ASTM D2734-91, 1991

[cit57] ASTM D790-10, 2010

[cit58] Bilisik K. , Karaduman N., Erdogan G., Sapanci E. and Gungor S., 13th Nanoscience and Nanotechnology Conference (NANOTR-13), TR, 295, 22–25 Oct 2017

